# Logical Intelligence and Mathematical Competence Are Determined by Physical Fitness in a Sample of School Children

**DOI:** 10.3389/fpsyg.2022.833844

**Published:** 2022-05-12

**Authors:** José Bracero-Malagón, Rocío Juárez-Ruiz de Mier, Rafael E. Reigal, Montserrat Caballero-Cerbán, Antonio Hernández-Mendo, Verónica Morales-Sánchez

**Affiliations:** ^1^University of Málaga, Málaga, Spain; ^2^Department of Evolutionary Psychology and Education, University of Málaga, Málaga, Spain; ^3^Department of Social Psychology, Social Work, Anthropology and East Asian Studies, University of Málaga, Málaga, Spain; ^4^Department of Physical Education, EADE Center, University of Wales Trinity Saint David, Málaga, Spain

**Keywords:** physical fitness, logical–mathematical intelligence, mathematical competence, preadolescence, exercise

## Abstract

Previous research has shown positive relationships between fitness level and different cognitive abilities and academic performance. The purpose of this study was to explore the relationships between logical–mathematical intelligence and mathematical competence with physical fitness in a group of pre-adolescents. Sixty-three children (50.79% girls; 49.21% boys) from Castro del Río (Córdoba, Spain), aged between 11 and 12 years (*M* = 11.44, *SD* = 0.64), participated in this research. The Superior Logical Intelligence Test (SLIT) and the EVAMAT 1.0–5 battery were used. Physical fitness was evaluated by the horizontal jump test, the 4×10 meter speed–agility test, and the Course Navette test. The analyses showed positive relationships between physical fitness with logical–mathematical intelligence and mathematical competence. Specifically, linear regression analyzes indicated that the 4×10 speed–agility test significantly predicted mathematical competence (*R*^2^ = 0.16; *β* = −0.41) and the horizontal jump test significantly predicted logical–mathematical intelligence (*R*^2^ = 0.24; *β* = 0.50). These results are in agreement with previous research, highlighting the importance of improving physical fitness from an early age due to its benefits for intellectual and academic development.

## Introduction

Physical exercise provides many health benefits. Among them, it reduces the risk of metabolic or cardiovascular diseases (Fiuza-Luces et al., 2018; Thyfault and Bergouignan, 2020), works as a protective factor against neurodegenerative processes (Whitty et al., 2020), strengthens the immune system (Holmen et al., 2020) or contributes to better cognitive development in childhood and adolescence (Sember et al., 2020). In contrast, sedentary lifestyles promote poorer health and increase the risk of disease (Narici et al., 2021). In fact, a sedentary lifestyle is considered one of the most important risk factor for mortality in the world. However, in recent decades there has been an evolution of lifestyles toward more sedentary habits (Bull et al., 2020).

In recent years, the impact that physical exercise can have on the brain in children has received special attention (Meijer et al., 2020). Structural and functional improvements have been observed in children who were regularly active (Valkenborghs et al., 2019). Physical exercise increased the blood flow, the synaptic transmission, and brain plasticity, promoting the increase in the volume of gray and white brain matter (Erickson et al., 2015; Bidzan-Bluma and Lipowska, 2018). Thus, a better development of cognitive abilities, such as attention, memory, or executive functions, has been observed (Samuel et al., 2017; Xue et al., 2019). This is relevant, given the impact that these capacities have on the adaptation and development processes at this stage of life. Specifically, these benefits in cognitive functioning can be extended to an essential issue for the development of children, such as competence and academic performance (de Greeff et al., 2018; Singh et al., 2019).

The practice of physical exercise has been shown to be important for cognitive health and could be related to the development of academic competencies, so that the Physical Education should be a subject with great importance in schools curriculum (Bailey, 2018). Especially due to the high rates of sedentarism that children and adolescents have currently (Gómez et al., 2020). Since many children are inactive in their daily life, practicing physical exercise in schools is of special interest to develop motor skills and raise awareness about active lifestyles (Hulteen et al., 2018). In this way, different intellectual and academic competences could improve due to physical exercise and the optimization of cognitive functioning derived from this process. For example, previous studies have highlighted the positive relationship between physical exercise and academic performance in disciplines, such as mathematics or reading comprehension (Hillman et al., 2009; Donnelly et al., 2016; Geertsen et al., 2016; Singh et al., 2019).

In addition, research published in recent years has shown that physical exercise must be carried out regularly and at moderate–high or high intensities to produce the greatest effects on cognitive functioning and intellectual abilities (Moreau et al., 2017; Reloba et al., 2017; de Greeff et al., 2018; Antunes et al., 2020). In fact, physical exercise must cause an increase in fitness level to adequately predict its effects on brain function (Chaddock et al., 2012, 2013; Esteban-Cornejo et al., 2019; Hernández-Mendo et al., 2019). Thus, various studies have shown positive relationships between aerobic capacity, strength, or speed–agility on attention, executive functioning, or academic performance (Donnelly et al., 2016; Singh et al., 2019; Solis-Urra et al., 2019; Páez-Maldonado et al., 2020).

Academic skills are the abilities that enable students to be successful in an academic context. Therefore, improving them is a necessity for the integral development of students. Specifically, learning mathematics is a challenge for children and adolescents due to its complexity and difficulty, but it is essential knowledge for their lives (Matthews, 2018). Therefore, it is relevant that scientific evidence suggests that promoting physical exercise at these ages could contribute to developing mathematical competencies (Bugge et al., 2018). As previously stated, the regular practice of exercise improve cognitive functioning. And according to Geertsen et al. (2016), better cognitive functions are positively correlated with the performance of a higher academic performance in the area of mathematics. Specifically, Chaddock et al. (2015) conducted a study with children between nine and ten years old, reported that a better physical fitness showed a statistically significant and positive relationship with arithmetic performance.

Traditionally, the learning of mathematics has been linked to logical–mathematical intelligence. This ability allows solving mathematical problems, which is based on reasoning, deduction, and abstract thinking (Cerda et al., 2011). This type of intelligence requires the development of higher order cognitive processes, called metacognitive skills, such as planning, control, selection, and evaluation of the process used in solving a task, which enables people to perform better in the area of mathematics and can predict academic performance (Ferrándiz et al., 2008; Contreras et al., 2009; Kroesbergen et al., 2009; Cerda et al., 2011).

Due to the importance of the development of mathematics at these ages, and the few investigations that have highlighted the associations between physical fitness with logical–mathematical intelligence, and mathematical performance together, the objective of this study was evaluate the relationships between physical fitness, logical–mathematical intelligence and mathematical competence in children between 11 and 12 years old.

## Materials and Methods

### Design

The study design was associative, comparative, and predictive (Ato et al., 2013).

### Participants

Sixty-three children (50.79% girls; 49.21% boys) from Castro del Río (Córdoba, Spain), aged between 11 and 12 years (M = 11.44, SD = 0.64). All the children were in fifth and sixth grade. The sampling was incidental, non-probabilistic. Students with physical health problems, who had not presented the informed consent signed by their parents, who were not 11 or 12 years old, or had any pathology that could affect the result of the investigation were excluded.

### Instruments

#### ALPHA-Fitness Battery

This battery was developed for the evaluation of health-related physical fitness in children and adolescents (Ruíz et al., 2011). the Horizontal Jump Test, the 4×10 Speed–Agility Test, and the Course Navette Test were implemented. For the Course Navette test (Léger et al., 1984), portable audio equipment and USB memory device were used. The initial speed of this test is 8.5 km/h, which has an increase of 0.5 km per minute during the course of the test. From the last stage completed in this test and the age of the subject, the maximum oxygen consumption (VO2max) was estimated indirectly using the formula (Léger et al., 1988): VO2max = 31.025 + (3.238 × V) - (3.248 × A) + (0.1536 × V × A); A = Age; V = Velocity (km/h). The protocol determined in the ALPA-Fitness Battery (Ruíz et al., 2011) was followed throughout the process.

#### Superior Logical Intelligence Test

The purpose of this test is to evaluate the inductive logical thinking of schoolchildren aged 11 years or older, a stage that coincides with the period of formal operations described by Piaget (Gray, 1990; Cerda et al., 2010). The SLIT has 50 figurative items to be solved, including abstract geometric shapes, such as points, lines, or polygons, among others. Each item has the same structure: left sector, 4 figures of a series united by a rule or pattern; right sector, 5 possibilities among which is the figure that completes the sequence on the left. The subject has to mark the figure on the right that continues the sequence on the left. In addition, at the beginning of the document, the test has 5 examples that would be solved with the help of the researcher in charge of administering the test to facilitate the understanding of the test, together with some indications on how to develop it successfully. The time required for the administration of this test is 30 min. Cerda et al. (2010) provide in the application manual of this test some guidelines for the correction of each item, being the maximum score of 50 points with a value of 1 point per correct answer and no penalty for errors or omissions. This instrument has an internal consistency of 0.95, calculated with Cronbach’s Alpha coefficient, being considered highly adequate.

#### EVAMAT 1.0 Battery

This test is an instrument for the assessment of the level of Basic Mathematical Competence that is expected to be developed during the fifth year of Primary Education, therefore, it can be applied both to subjects in the fifth year and to subjects in the sixth year, from 11 to 12 years of age (García-Vidal et al., 2011). This battery collects information on five dimensions of mathematical competence: (1) Numeration; (2) Calculation; (3) Geometry and Measurement; (4) Information and Chance; (5) Problem-solving. Each dimension is made up of a series of tasks to be carried out by the students, which are accompanied by instructions that facilitate their understanding together with solved examples. For the correction of this battery, García-Vidal et al. (2011) provide guidelines for correction and interpretation, which allow to obtain the scores obtained by the students partially in each dimension of the test and globally, for which it is necessary to follow the formulas given to obtain these scores. Depending on the degree of difficulty of the task, it will have a certain score for the correct answers, being penalized the errors and omissions in some of them. This battery has a reliability of 0.96, therefore, it is considered highly adequate.

### Procedure

The sample was selected in different schools in the city of Castro del Río, Córdoba (Spain). First, the director of the school where the study was carried out was contacted by telephone. The objective of the investigation was explained and permission was requested to carry it out. Once the participation was accepted, the informed consent of the parents or legal guardians was obtained. In addition, the ethical principles of the Declaration of Helsinki (World Medical Association, 2013) were respected and it was approved by the Ethics Committee of the University of Malaga (No. 243, CEUMA Registry No. 18-2015-H).

During the first half of April 2021, the ALPHA-Fitness battery was implemented. The physical fitness tests were carried out in the sports facilities of the school, from 10 to 12 h in the morning. Prior to its application, an eight-minute warm-up was performed based on low intensity continuous running, joint mobility, and dynamic stretching. The order of application of the different tests was: (1) Horizontal Jump Test; (2) Speed–Agility Test 4 × 10 meters; and (3) Course Navette Test. The logical–mathematical competence tests, SILT and EVAMAT 1.0, were applied during the second half of April. Both instruments were clearly explained so that there were no doubts.

### Data Analysis

Descriptive and inferential analyses were performed. The Kolmogorov–Smirnov test was applied to analyze normality. Correlations were assessed with the Pearson coefficient (±0.01 to ±0.19 = very weak correlation; ±0.20 to ±0.39 = weak correlation; ±0.40 to ±0.59 = moderate correlation; ±0.60 to ±0.79 = high correlation; Evans, 1996). The predictive capacity of the physical fitness over the other variables was verified by means of linear regression analysis (successive steps). The level of significance was set to α = 0.05. Analysis of variance components and generalizability analysis have also been performed to show that the sample is reliable and the results are generalizable. The software SPSS v.20.0 (IBM Corp., Armonk, NY, United States) was used for statistical processing.

## Results

Table 1 shows the descriptive and normality statistics of the variables of physical fitness, logical–mathematical intelligence, and mathematical competence. As observed, some variables did not show a normal distribution (*p* < 0.05). To adjust these distributions, the algorithms l (x), x2, and ln (x) were used.

**Table 1:** Descriptive measures, skewness, kurtosis, and Kolmogorov–Smirnov test for study variables.

	*M*	*SD*	*S*	*K*	*K–S*
**EVAMAT 1.0**					
EVAMAT	164.87	37.73	−0.86	0.87	0.02*
Numeration	34.59	6.87	−1.30	1.63	0.20
Calculation	32.99	8.35	−1.05	1.46	0.20
Geometry and measurement	32.45	8.15	−0.46	−0.55	0.01*
Information and chance	32.16	6.84	−1.38	2.60	0.20
Problem-solving	32.84	15.94	−0.23	−0.55	0.20
**Superior Logical Intelligence Test (SLIT)**					
Logic	22.87	8.14	0.34	−0.99	0.06
**Alpha fitness**					
Horizontal jump test (cm)	138.71	19.55	0.39	−0.20	0.02*
4×10 speed–agility test (sec)	13.26	1.48	0.99	0.90	0.00*
VO2max (ml/kg/min)	40.99	4.15	1.06	0.70	0.07*

*M = Mean; SD = Standard Deviation; S = Skewness; K = Kurtosis; K–S = Kolmogorov–Smirnov*.

* *p < 0.05.*

Table 2 shows the correlations between physical fitness, logical–mathematical intelligence and mathematical competence. The Horizontal Jumping Test shows a statistically significant correlation with EVAMAT (*r* = 0.26, *p* < 0.05), Problem-Solving (*r* = 0.33, *p* < 0.01) and Logic (*r* = 0.43, *p* < 0.01). Therefore, the Motor Ability variable presents statistically significant correlations with Numeration (*r* = −0.29, *p *< 0.05), Logic (*r*  =−0.31, *p* < 0.05) and EVAMAT (*r* = −0.41, *p* < 0.01), Calculation (*r* = −0.37, *p* < 0.01) and Problem-Solving (*r* = −0.42, *p* < 0.01). The Maximum Oxygen Consumption shows a statistically significant correlation with EVAMAT (*r* = 0.27, *p* < 0.05) and Problem-Solving (*r* = 0.30, *p* < 0.05). Also, EVAMAT present a statistically significant direct linear correlation with the variable Logic, highlighting the significance of EVAMAT at the 0.01 level (*r* = 0.60, *p* < 0.001) and Problem-Solving at the same level (*r* = 0.57, *p* < 0.001).

**Table 2:** Correlations between the variables of logical–mathematical performance and physical fitness.

	Horizontal jump test (cm)	4×10 Speed–agility test (s)	VO2max (ml/kg/min)	Logic
EVAMAT 1.0	0.26*	−0.41**	0.27*	0.60**
Numeration	0.16	−0.29*	0.20	0.37**
Calculation	0.08	−0.37**	0.24^a^	0.41**
Geometry and measurement	0.17	−0.22	0.18	0.43**
Information and chance	−0.001	−0.15	0.12	0.39**
Problem-solving	0.33**	−0.42**	0.30*	0.57**
Logic	0.43**	−0.31*	0.10	−

^a^*p* = 0.06.

* *p < 0.05;*

** *p < 0.01*.

Table 3 shows the linear regression analyses (stepwise) with which we attempted to identify the physical fitness variables that predict the values of logical–mathematical intelligence and mathematical competence. The models meet the assumptions of linearity in the relationship between predictor variables and criteria, homoscedasticity, and normal distribution of residuals whose mean value is 0 with a standard deviation of almost 1 (0.99). Variables not included in the different models were excluded due to lack of significance (*p* > 0.05). The Durbin–Watson value was between 1.62 and 2.16, which is appropriate according to Pardo and Ruiz (2005), indicating that it can be assumed that the residuals are independent and the assumption of independence of the independent variables with respect to the dependent variable is fulfilled.

**Table 3:** Linear regression analysis.

R	R^2^ adj.	D–W	Criterion	Predictor	Beta^a^ (CI 95%)	Beta^b^	*t*	*T*	*FIV*
0.41	0.16	1.81	EVA	4×10 Test	−43.01 (−67.68, −18.33)	−0.41	−3.49*	1.00	1.00
0.29	0.07	2.16	NUM	4×10 Test	−17.50 (−32.78, −2.22)	−0.29	−2.29*	1.00	1.00
0.37	0.12	1.63	CAL	4×10 Test	−28.10 (−46.57, −9.64)	−0.37	−3.05*	1.00	1.00
0.42	0.16	1.62	PRO	4×10 Test	−60.68 (−95.18, −26.17)	−0.42	−3.52*	1.00	1.00
0.50	0.24	1.78	LOG	HJT	0.22 (0.11, 0.35)	0.50	3.83**	1.00	1.00

*EVA = EVAMAT; NUM = Numeration; CAL = Calculation; PRO = Problem-solving; LOG = Logic; HJT = Horizontal jump test; D–W = Durbin–Watson; Beta^a^ = unstandardized; Beta^b^ = standardized T = Tolerance; FIV = Variance inflation factor;*

* *p < 0.05;*

** *p < 0.01*.

As observed, analyses indicate that the 4 × 10 speed–agility test significantly predicts scores for EVAMAT (*R* = 0.41; corrected *R*^2^ = 0.16; *F* = 12.16; *p* < 0.05), Numeration (*R* = 0.29; *R*^2^ corrected = 0.07; *F* = 5.25; *p* < 0.05), Calculation (*R* = 0.37; *R*^2^ corrected = 0.12; *F* = 9.27; *p* < 0.05) and Problem-Solving (*R* = 0.42; *R*^2^ corrected = 0.16; *F* = 12.38; *p* < 0.05). In addition, the Horizontal Jump Test predicted the Logic values (*R* = 0.50; corrected R^2^ = 0.24; *F* = 14.64; *p* < 0.001).

## Discussion

The purpose of this study was to analyze how physical exercise and physical fitness are related to logical–mathematical intelligence and mathematical competence in children between 11 and 12 years old. The results obtained showed statistically relationships between the measures studied and, therefore, the objective of the research is achieved. Specifically, the data reveal positive relationships between physical performance and the logical–mathematical competence variables analyzed. This is congruent with other research that had pointed out positive relationships between physical condition and mathematical performance at these ages (Chaddock et al., 2015; Geertsen et al., 2016).

This research focuses on very important parameters of intellectual and academic competence at these ages. Specifically, the logical–mathematical competence has been explored, represented by the variables of logical–mathematical intelligence, measured with the Superior Logical Intelligence Test (SLIT), and Basic Mathematical Competencies, evaluated with the EVAMAT 1.0 test (which contains the subtests: Numeration, Calculation, Geometry and Measurement, Information and Chance and Problem-solving). Developing these skills in childhood is very relevant. First, because of their own difficulty. Therefore, if physical exercise and increased physical performance can promote their learning, it is an important matter. Second, because of the impact it can have on other academic competencies throughout the student’s life (Ferrándiz et al., 2008; Contreras et al., 2009; Kroesbergen et al., 2009; Cerda et al., 2011; Matthews, 2018).

The results show that those students with high logical intelligence perform better in the area of Mathematics, showing a greater correlation with problem-solving. Thus, the results coincide with the evidence of other authors regarding the relationship between logical intelligence and different parameters of mathematical performance (Cerda et al., 2011). But in addition, and this research focuses on this, it has been observed that the evaluated physical fitness (strength, speed–agility, and maximum oxygen consumption) have maintained statistically significant correlations with all the logical–mathematical competence variables, except with Geometry and Measurement, as well as Information and Chance. These results are consistent with previous studies that have shown that exercise and physical fitness was related to better cognitive development in childhood (Samuel et al., 2017; Bidzan-Bluma and Lipowska, 2018; Xue et al., 2019; Sember et al., 2020), and, specifically, to a more efficient development of basic academic competences, such as mathematics, among others (Hillman et al., 2009; Donnelly et al., 2016; Geertsen et al., 2016; Singh et al., 2019).

In this study, the speed–agility and the horizontal jump test have predicted the EVAMAT, Numeration, Calculation, Problem-Solving, and Logic scores. Additionally, maximal oxygen consumption has been correlated with EVAMAT and Problem-Solving scores. This suggests that increasing the level of physical performance is a necessary process for the development of these competencies. In previous studies, the variable that best predicted cognitive and intellectual functioning was cardiorespiratory fitness in children and adolescents. However, in this research, speed–agility and strength have been more decisive. This suggests that other types of fitness parameters could offer relevant information on how physical exercise is related to brain function. In fact, although cardiorespiratory fitness is traditionally associated with better brain health, various studies have also shown that speed or strength over parameters with good predictability (Donnelly et al., 2016; Pérez-Lobato et al., 2016; Domínguez-González et al., 2018; Singh et al., 2019; Solis-Urra et al., 2019; Páez-Maldonado et al., 2020). In any case, and as a general indication, it is clearly established that physical fitness is a good indicator of cognitive and intellectual functioning (Chaddock et al., 2012, 2013; Moreau et al., 2017; Reloba et al., 2017; de Greeff et al., 2018; Esteban-Cornejo et al., 2019; Antunes et al., 2020).

The increase in logical–mathematical intelligence and mathematical competences would be favored by the development of cognitive functions, such as cognitive flexibility, working memory, and sustained attention (Geertsen et al., 2016; Egger et al., 2019). In addition, authors such as Chaddock et al. (2015) or Esteban-Cornejo et al. (2019) have pointed out that physical exercise and a better physical condition facilitate positive changes in the volume of gray and white matter in cortical and subcortical areas that would be linked to these mathematical processes. These changes would aid in more effective math learning and overall academic achievement. There are multiple hypotheses put forward for the explanation of these relationships, with some of those put forward by experts being the increase in cerebral blood flow or the release of neurotrophins, such as BDNF or IGF1, which contribute to these neurological modifications (Hernández-Mendo et al., 2019).

This study has some limitations. Firstly, increasing the sample would be an aspect that could clarify the relationships extracted in this study, in addition to being able to delve deeper into how other variables, such as gender or age, affect the study. The pandemic situation has not helped or facilitated the increase in the sample, in addition to impairing the fluidity in the execution of the research, since the application of the questionnaires has been delayed due to contagion among the students. As this is a comparative study, it has not been possible to establish causal relationships between the results obtained, so that in future research it would be interesting to carry out experimental or longitudinal designs to analyze more accurately the impact generated by the physical fitness on the variables under study. The search for the most effective methodology for the implementation of physical activity programs with which to improve the physical fitness of schoolchildren, with the aim of obtaining the maximum possible benefits in logical–mathematical competence would be a suggestive purpose for future research, in which different exercise programs would be applied. Finally, only the level of physical fitness has been evaluated. It would be interesting in future studies to observe how other factors, such as rest time, diet, or study hours, could be modulating the relationship between physical fitness and academic skills.

The results shown in this research contribute to reinforce the conclusions of previous research on the relationship between physical fitness and academic performance, justifying the promotion of the practice of physical activity from an early age. Therefore, professionals and organizations closely related to children should take into account the contributions made by this type of research to focus education toward active lifestyles that make it possible to achieve the benefits associated with them, for the achievement of an integral development of society. Specifically, this study concludes that a good physical fitness enhances benefits in variables related to logical–mathematical competence, which can have a great impact on the development of children and adolescents.

## Data Availability Statement

The raw data supporting the conclusions of this article will be made available by the authors, without undue reservation.

## Ethics Statement

The studies involving human participants were reviewed and approved by Ethics Committee of the University of Málaga (No. 243, CEUMA Registry No. 18-2015-H). Written informed consent to participate in this study was provided by the participants’ legal guardian/next of kin.

## Author Contributions

JB-M, RJ-RM, RR, MC-C, AH-M, and VM-S participated in the study design and data collection, performed statistical analyses and contributed to the interpretation of the results, wrote the manuscript, approved the final manuscript as submitted, and reviewed and provided feedback to the manuscript. All authors contributed to the article and approved the submitted version.

## Conflict of Interest

The authors declare that the research was conducted in the absence of any commercial or financial relationships that could be construed as a potential conflict of interest.

## Publisher’s Note

All claims expressed in this article are solely those of the authors and do not necessarily represent those of their affiliated organizations, or those of the publisher, the editors and the reviewers. Any product that may be evaluated in this article, or claim that may be made by its manufacturer, is not guaranteed or endorsed by the publisher.
